# Factors impacting resident outcomes from COVID-19 outbreaks in Residential Aged Care Facilities in Sydney Local Health District: testing an infection prevention and control scoring system

**DOI:** 10.1186/s12889-023-16634-3

**Published:** 2023-09-11

**Authors:** Alison Stubbs, Elizabeth Dawson, Elise Campbell, Joseph Van Buskirk, George Johnson, Natasha Spalding, John Cullen, Karen Chee, Jodi McLeod, Luke D. Knibbs, Jodie O’Callaghan, Christian Jones, Chinonye Maduka, Patricia Fleming, Reuben Haupt, Andrew Penman

**Affiliations:** 1 Sydney Local Health District Public Health Unit, Sydney, NSW Australia; 2 Sydney Local Health District, Infection Prevention and Control, Sydney, NSW Australia; 3 Sydney Local Health District Residential Aged Care Facility Outreach Team, Sydney, NSW Australia; 4 School of Public Health, University of Sydney, Sydney, NSW Australia; 5 Sydney Local Health District Executive, Sydney, NSW Australia

**Keywords:** COVID-19, Outbreaks, Aged care facilities, Infection prevention and control

## Abstract

**Background:**

COVID-19 outbreaks have disproportionately affected Residential Aged Care Facilities (RACFs) around the world, with devastating impacts for residents and their families. Many factors such as community prevalence, facility layout, and infection control practices have been linked to resident outcomes. At present, there are no scoring systems designed to quantify these factors and assess their level of association with resident attack rates and mortality rates.

**Methods:**

We constructed a novel Infection Prevention and Control (IPC) scoring system to quantify facility layout, ability to cohort residents, and IPC practices in RACFs. We conducted a retrospective observational cohort study of COVID-19 outbreaks, applying our IPC scoring system to all COVID-19 outbreaks occurring in RACFs in Sydney Local Health District during the Delta and Omicron waves of the COVID-19 pandemic in New South Wales, Australia.

**Results:**

Twenty-six COVID-19 outbreaks in 23 facilities in the Delta wave, and 84 outbreaks in 53 facilities in the Omicron wave were included in the study. A linear Generalised Estimating Equation model was fitted to the Omicron data. Higher IPC scores were associated with higher attack rates and mortality rates. Facilities with IPC scores greater than 75.0% had attack rates 19.6% higher [95% CI: 6.4%-32.8%] and mortality rates 1.7% higher [95% CI: 0.6%-2.7%] than facilities with an IPC score of less than 60.0%.

**Conclusions:**

The results of this study suggest the utility of the IPC scoring system for identifying facilities at greater risk of adverse outcomes from COVID-19 outbreaks. While further validation and replication of accuracy is required, the IPC scoring system could be used and adapted to improve planning, policy, and resource allocation for future outbreaks.

**Supplementary Information:**

The online version contains supplementary material available at 10.1186/s12889-023-16634-3.

## Background

Coronavirus Disease-19 (COVID-19) is caused by infection with the novel Severe Acute Respiratory Syndrome Coronavirus 2 (SARS-CoV-2), first identified in humans in Wuhan, China in December 2019 [1]. Since it was first declared a global pandemic by the World Health Organisation (WHO) in March 2020, there have been over 660 million cases of COVID-19 and over 6.7 million COVID-19-related deaths worldwide [2].

Older people living in Residential Aged Care Facilities (RACFs) have been disproportionately affected by COVID-19 outbreaks, with numerous studies showing higher rates of infection, morbidity, and mortality compared with the general population [3–5]. In Great Britain, the 2021–22 COVID-19-related mortality rate among RACF residents was 4652 per 100 000, compared to 479 per 100 000 in community-dwelling older adults, with similar disparities in the USA, Norway and Sweden [6]. In Australia in 2020, seven percent of COVID-19 cases and 75% of related deaths occurred in RACF residents [7]. A systematic review of 49 studies of COVID-19 outbreaks in RACFs across 14 countries found that the pooled single-facility attack rate was 45% (95% CI: 32–58%) and the pooled case fatality rate was 23% (95% CI: 18–28%) [8]. The reasons for the profound impact of COVID-19 on aged care residents and their families are multifactorial. Factors which have been shown to increase the risks of COVID-19 transmission include communal living, the need for frequent close contact between residents and staff for personal care, and the difficulties of social distancing and adherence to guidelines for residents with cognitive impairment [9]. Building design, operation, and facility layout also contribute [10]. In addition, RACF residents have a higher prevalence of comorbidities than the general population, which increases the risk of COVID-related morbidity and mortality [11, 12].

Since the start of the COVID-19 pandemic, there have been numerous severe outbreaks reported in RACFs, both in Australia and overseas [5, 13]. The level of community transmission has been shown to be the most consistent predictor of COVID-19 outbreaks in RACFs [9], with facilities in urban areas with higher community COVID-19 prevalence associated with higher rates of COVID-19 outbreaks [14, 15]. Facility size has also been linked to COVID-19 outbreaks in RACFs, with larger facilities with higher numbers of residents at greater risk, possibly due to higher numbers of staff and visitors increasing the risk of COVID-19 incursions [5, 9]. While some studies have suggested a link between measures of facility quality, such as the Nursing Home Compare 5-star ratings in the USA, and COVID-19 outbreaks [16, 17], a systematic review including 16 studies which examined the relationship between the 5-star rating system and COVID-19 outcomes found no association between them [9].

There are fifteen Local Health Districts (LHDs) in the Australian jurisdiction of New South Wales (NSW). In 2021, the population of Sydney LHD (SLHD), was estimated at over 720 000, with 9.7% of the population over the age of 70 [18]. There are 56 RACFs located within SLHD. The SLHD Public Health Unit (PHU) and the SLHD RACF Outreach team, which provided clinical input for RACF residents, worked closely with the 56 RACFs to manage COVID-19 cases using a colour-coded lockdown system. ‘Red’ lockdowns were implemented in response to a COVID-19 outbreak (i.e., one or more COVID-19 positive residents, staff, or visitors in an RACF). Our study investigated factors impacting resident outcomes from ‘red’ lockdowns occurring during the most significant waves of the pandemic for RACFs in NSW, Delta and Omicron.

The aim of this study was to test a scoring system to explore and quantify the Infection Prevention and Control (IPC) related factors impacting on COVID-19 outbreaks in SLHD RACFs, and to determine if the IPC scores were specifically associated with higher resident attack and mortality rates.

## Methods

We conducted a retrospective, observational cohort study of all COVID-19 outbreaks occurring in RACFs in SLHD from 16 June 2021 to 28 February 2022. This period was chosen because it spans the Delta and Omicron waves of the pandemic, from the first case of Delta notified in NSW on 16 June 2021 [19], to the end of February, when Omicron cases in the community began to decrease [20]. Twenty-seventh November 2021 was considered the start of Omicron, when the first case was detected in NSW [21]. We designed two scoring systems to quantify the quality of IPC practices and management of the facilities. Ethics approval was granted by SLHD Human Research Ethics Committee (2022/ETH00754).

### Population

All 56 RACFs within SLHD were included in this study. The unit of analyses was individual ‘red’ lockdowns, which occurred in response to a COVID-19 outbreak in a facility. During the Delta wave, an outbreak was defined as a single case of COVID-19 in a resident, staff member, or visitor of an RACF. During Omicron, an outbreak was defined as a single resident testing positive to COVID-19 on a polymerase chain reaction (PCR) test, or a staff member or visitor testing positive by PCR with high-risk exposure to residents during their infectious period.

### Data sources

The data used for this project were originally collected for the purpose of the management of COVID-19 outbreaks and were spread across various sources held by the PHU and RACF Outreach teams. These included IPC review reports, SLHD’s Executive Outbreak Management Team (OMT) meeting minutes, handover documents, line lists and emails.

### Data extraction process

The data sources were retrospectively searched during June to August 2022, and the relevant data were extracted and entered into Research Electronic Data Capture (REDCap), a secure web application for building and managing electronic databases [22, 23]. Uniform training of data collectors, and a standard operating procedure (SOP) document were used to ensure consistency in the data extraction process.

Two scoring systems, the IPC scoring system, and the Management scoring system, were designed specifically for this project. The IPC scoring system was developed based on reviews of RACFs conducted by the RACF Outreach team. It quantified factors that may impede the control of an outbreak into three sections using the matrix in Appendix A. The ‘RACF layout evaluation’ section of the IPC scoring system included measures of facility layout, for example, bed to bathroom ratio. The ‘IPC evaluation by zone’ section measured how effectively each zone within the facility could be contained. This included factors such as whether there was a separate entrance and exit for each zone, and whether there was a nursing station within the zone. The’IPC issues by zone’ section measured IPC factors in each zone. These included staff Personal Protective Equipment (PPE) and hand hygiene compliance, PPE supply, donning and doffing stations, cleaning and waste management, staff and resident swabbing, ventilation, signage, and staffing. The scoring matrix was designed to list each IPC related factor that was assessed during inspections conducted by the RACF outreach team. These data were available in IPC review reports, which documented these inspections. Using the IPC scoring matrix, each factor was given a score, and these were added to give a cumulative total. The numerical values assigned to each variable were based on an assessment of risk made by the RACF outreach team. Within each variable, factors deemed highest risk for an outbreak were given the highest score. For example, for the variable number of rooms, > 100 was deemed highest risk and was given a score of 2, and < 100 was deemed lower risk and was given a score of 1. Two experienced researchers from the RACF Outreach team completed the scoring matrix independently for each facility. The results were then compared, and discrepancies resolved by discussion, to give the final IPC score for each facility, which was then converted to a percentage for the analysis. Higher scores indicated a perceived higher risk of a COVID-19 outbreak and poorer resident outcomes.

The Management scoring system was designed to rate the quality of management of each facility, using the matrix in Appendix B. It quantified the level of concern among SLHD staff regarding facility management. The level of concern was based on a comprehensive assessment by the research team of key qualitative aspects of RACF management, including preparedness, education and understanding, responsiveness, support, and communication. Two independent members of the research team, each with extensive experience dealing with facility management during the pandemic, independently assigned a score to each facility ranging from 0 to 4, with 0 indicating no concerns with the facility management, and 4 indicating urgent concerns. The two scores were compared and in the case of any discrepancies, a third experienced member of the research team assigned a final deciding score, which was used in the analysis.

### Data analysis

The outcomes of interest were resident attack rate and resident COVID-19 related mortality rate. Attack rate was calculated as the number of residents who tested positive to COVID-19 during an outbreak, as a percentage of the number of residents in the facility at the start of the outbreak. Mortality rate was the number of COVID-19 related resident deaths during the outbreak, also as a percentage of the number of residents in the facility at the start of the outbreak.

All available variables were assessed by the research team for a plausible association with the outcomes of attack rate and mortality rate that, if confirmed, could have meaningful implications for policy and management of future outbreaks. Variables relating to dates of outbreaks and IPC reviews, staff vaccination and staff COVID-19 positivity rates were not included in the analysis due to high rates of missing data or insufficient variability. In addition, State Government guidelines changed during the study period regarding the duration of lockdowns and staff vaccination requirements. As such, the duration of outbreaks and rates of staff vaccination could not be directly attributed to the facility. The outcome variable of hospitalisations was also excluded due to missing data, and difficulty determining the reason for hospitalisation (whether due to the severity of COVID-19 or another reason).

Based on the assessment of the research team, the following exposure variables were selected for analysis: IPC score, Management score, number of residents, proportion of residents with a single room, and proportion of residents up-to-date with vaccinations. For the purposes of this project, ‘up-to-date’ was considered to be 2 doses of COVID-19 vaccine received before the start of the outbreak during Delta, and 3 doses received before the start of the outbreak during Omicron. There were three missing values (3.6%) for proportion of residents up-to-date with vaccinations and these were excluded from the analysis. Data were complete for all other variables.

Due to the skewness and clustering of the data, variables were categorised for the statistical analysis. Categorisations were informed by tertiles, which were amended slightly in the interest of intuitive and interpretable categories. Using the statistical software R, (version 4.1.1) [24] linear generalised estimating equations (GEE) were fitted to the data using the ‘gee’ package (version 4.13.24) [25] to control for autocorrelation of observations from the same facility. An unstructured correlation structure was adopted, although sensitivity analyses revealed comparable results using other correlation structures. Sensitivity analyses were also conducted where variables were fit continuously, with non-linear functions (both natural splines and quadratic terms) to account for deviations from linearity, with these models yielding comparable conclusions to when the exposures were categorised. In the interest of interpretability, models with categorised covariates were chosen as the final models.

## Results

There are 56 RACFs in SLHD and all were considered for inclusion in this study. One facility did not have any red lockdowns during the study period and was excluded. Fifty-five RACFs were included in the analysis. Delta and Omicron outbreaks were separated for analysis, due to differences in outbreak definitions, management practices, and data availability between the two waves.

### Summary of outbreaks

Detailed descriptive statistics summarising all outbreaks occurring during Delta and Omicron are presented in Table 1, below. In summary, thirty outbreaks were notified during the Delta wave (16 June 2021 – 26 November 2021). Of these, three were excluded from the final analysis because they did not meet the definition of a ‘red’ lockdown, and a further one was excluded as no data were available. Among the Delta outbreaks, the number of missing values for each variable was high, up to 18 for one exposure variable. Due to the amount of missing data, the low number of outbreaks, cases, and deaths, only descriptive statistics are reported for the Delta outbreaks.

**Table 1:** Summary of exposure and outcome variables for COVID-19 outbreaks in SLHD RACFs

	**Delta**	**Omicron**
**Number of outbreaks**	26	84
**Number of facilities affected**	23	53
**Mean number of outbreaks per facility**	1.1 (SD 0.5)	1.6 (SD 0.7)
**Total COVID-19 positive cases (residents)**	63	702
**Total COVID-19 related deaths (residents)**	5	59
**Total COVID-19 cases (staff)**	25	885
**Exposure Variables**^a^	Mean (SD)	Mean (SD)
Number of residents	74.2 (33.1)	70.5 (30.2)
Residents with single room (%)	33.3 (45.7)	63.1 (39.1)
Residents with up-to-date vaccinations^b^ (%)	66.0 (28.5)	75.9 (27.9)
IPC^c^ combined score (%)	63.5 (13.0)	65.33 (13.4)
Management score	2.03 (1.3)	1.67 (1.2)
**Outcome Variables**	Mean (SD)	Mean (SD)
Attack rate (%)	7.8 (13.1)	12.4 (22.3)
Mortality rate (%)	0.6 (1.8)	1.00 (2.2)

^a^Exposure variables referring to residents show the mean total number of residents at each facility

^b^For the purpose of this project, vaccinations were considered ‘up-to-date’ if residents had received 2 doses before the start of the ‘red’ lockdown during the Delta wave, or 3 doses before the start of the ‘red’ lockdown during the Omicron wave

^c^Infection Prevention and Control

During the Omicron wave (27 November 2021 – 28 February 2022), there were 89 outbreaks notified across 53 facilities. Five Omicron outbreaks for which data were collected were excluded from the analysis for the following reasons: outside study period (*n* = 2), did not meet the red lockdown definition (*n* = 2), and missing outcome data (*n* = 1). Two RACFs did not have any red lockdowns during the Omicron wave, thus, 53 facilities were included in the Omicron data.

Omicron outbreaks were selected for further statistical analysis, due to the larger sample size and higher quality data compared to Delta. A linear GEE model was constructed to investigate potential associations between exposure and outcome variables, controlling for autocorrelation between repeat outbreaks within facilities.

Table 2, below, shows the results of this analysis, with mutually adjusted coefficients and 95% confidence intervals reported for all variables.

**Table 2:** Coefficients for attack and mortality rates during Omicron COVID-19 outbreaks in SLHD RACFs

**Variable**		**Attack Rate (%)**		**Mortality Rate (%)**	
	**Number of facilities**	**Mean [SD]**	**Coefficient [95% CI]**	**Mean [SD]**	**Coefficient [95% CI]**
Intercept			19.4 [6.11—32.7]**		1.42 [0.161—2.68]*
Number of residents					
**< 50** vs 50—75	16	10.4 [24.0]	0.7 [-14.8—16.2]	0.8 [2.6]	0.6 [-0.9—2.0]
**50–75**	22	17.2 [23.7]	Ref	1.2 [2.4]	Ref
**> 75** vs 50—75	17	9.2 [19.1]	3.6 [-10.9—18.1]	0.8 [1.8]	0.7 [-0.5—1.9]
Residents with single room					
**< 50%**	24	19.5 [29.4]	Ref	1.9 [3.1]	Ref
**50%—95%** vs < 50%	11	6.4 [11.4]	-12.6 [-27.4—2.2]	0.5 [1.5]	-1.4 [-2.6—-0.1]*
**> 95%** vs < 50%	21	8.5 [16.1]	-3.11 [-17.7—11.5]	0.3 [0.9]	-1.1 [-2.2—0.04]
Residents with up-to-date vaccinations					
**< 70%** vs 70%—95%	18	11.5 [19.6]	-12.6 [-24.8—-0.4]*	1.2 [2.4]	-0.4 [-1.6—0.7]
**70%—95%**	22	18.8 [27.5]	Ref	1.4 [2.7]	Ref
**> 95%** vs 70%—95%	17	7.8 [19.0]	-12.2 [-24.9—0.5]	0.4 [1.4]	-1.09 [-2.2—-0.02]*
Management Score 3–4 vs 0–2					
**0–2**	38	13.2 [23.4]	Ref	1.1 [2.4]	Ref
**3–4**	15	10.2 [19.2]	-8.05 [-19.1 – 3.0]	0.7 [1.6]	-0.9 [-1.9—0.2]
IPC Score					
**< 60%**	20	4.4 [7.7]	Ref	0.1 [0.5]	Ref
**60%—75%** vs < 60%	16	12.5 [22.8]	2.7 [-8.1—13.4]	1.0 [2.5]	0.3 [-0.6—1.3]
**> 75%** vs < 60%	17	23.5 [30.2]	19.6 [6.4—32.8]**	2.1 [2.9]	1.7 [0.7—2.7]**

NB: * *p* < 0.05; **p < 0.01; Ref denotes reference category

Results of the GEE analysis in Table 2 revealed varying levels of association between exposure and outcome variables. Facilities with IPC scores greater than 75.0% had attack rates 19.6% higher [95% CI 6.4–32.8] and mortality rates 1.7% higher [95% CI 0.7–2.7] than facilities with an IPC score of less than 60%. Facilities with an IPC score of 60.0–75.0% had similar attack and mortality rates to facilities with IPC scores of less than 60.0% (attack rate coefficient 2.7 [95% CI -8.1–13.4] and mortality rate coefficient 0.3 [95% CI -0.6 – 1.3]). The analysis also suggested an association between the proportion of residents in a single room, and outcomes, however the results for attack rate were not statistically significant. For facilities with 50.0–95.0% of residents in a single room, the attack rate coefficient was -12.6 [95% CI: -27.4 – 2.2] and the mortality rate coefficient was -1.4 [95% CI: -2.6- -0.1] compared to facilities with less than 50.0% of residents in a single room. Results for other variables such as number of residents, residents up-to-date with vaccinations, and Management scores showed less substantial differences in attack and mortality rates.

## Discussion

Understanding the factors that drive COVID-19 outbreaks in RACFs is crucial to make changes to public health policy and practice that will improve management of future outbreaks in the aged care sector. Many studies have attempted to explore relationships between individual characteristics of RACFs and COVID-19 outbreaks. Facility layout has been linked to COVID-19 outcomes in a meta-analysis of 41 studies [26], and an association between facility size and outcomes has been demonstrated in two systematic reviews [5, 9]. Our study proposed the use of an IPC scoring system to integrate multiple factors into a single score, and a Management scoring system to rate the quality of facility management. To the best of our knowledge, this is the first study to develop such scoring systems and assess their association with resident outcomes from observed COVID-19 outbreaks in RACFs.

The results of our analysis demonstrate the utility of the IPC score, in that higher IPC scores were associated with higher attack and mortality rates during COVID-19 outbreaks. While further validation of the IPC score and replication of its accuracy is required, this study provides preliminary evidence that the IPC scoring system may be a useful tool for identifying facilities at a greater risk of adverse outcomes. Refinement of the IPC scoring matrix is needed to ensure evidence-based weightings are applied to each element. After such development, there is potential for the IPC scoring system to be used prospectively to assess the preparedness of a facility to manage and attenuate the impacts of COVID-19 during an outbreak. This would allow identification and targeted resourcing of facilities at higher risk of poor outcomes.

We were not able to demonstrate a link between the facility Management score and the outcomes of attack or mortality rate, suggesting that the Management scoring system is not a useful predictor of outcomes. In the literature, attempts to link other measures of RACF quality, such as the 5-star rating system, to COVID-19 outcomes have produced inconsistent results [16, 17]. Further work is needed to explore the relationship between quality indicators and COVID-19 outcomes in RACFs.

Our data suggests that facilities with a higher proportion of residents in a single room had better outcomes, in keeping with findings from the literature [10]. However, number of residents in a facility did not appear to influence attack rate or mortality rate after accounting for all other covariates, which contrasts with evidence suggesting that larger facility size is associated with poorer outcomes [5, 9]. The proportion of residents up-to-date with vaccinations also produced unexpected results; with the mean attack rate being highest for the middle category of 70.0–95.0% residents up-to-date with vaccination. These unanticipated results may be due to the influence of the limitations discussed below.

This study is limited by relatively poor data quality, as documentation was originally for the purposes of outbreak management rather than research. The research team were unable to elicit data for variables such as reasons for hospitalisation, and such variables were therefore excluded from the analysis. Despite having two independent reviewers assigning scores for the IPC and management scoring systems, with discrepancies resolved by discussion (IPC) or a third reviewer (Management), both scores remain somewhat subjective. The IPC score did not take into account the contribution of effect of each variable. Variables with multiple categories had a wider range of scores than binary variables, yet this was unrelated to the level of risk. Variables with the same score did not necessarily contribute equally to the risk. Only COVID-19 cases and deaths that occurred during the lockdown period were included in this study. This may partly explain why the mean attack rate and mortality rate for both Delta and Omicron data in our study were lower than in the literature [4, 8]. Our sample size was small, thus, the power to detect associations was low, and confidence intervals were wide. Due to the retrospective cohort design, the risk of confounding and bias in our study is high. While we attempted to collect data on potential confounders such as the timing of IPC reviews, exact dates of vaccinations, and duration of outbreak, these variables could not be included in the analysis due to the proportion of missing data. It is also important to note that some elements of the IPC score, such as number of rooms, correlated with other variables in the model, such as number of residents. Recall bias may have affected the Management score, which was assigned retrospectively after the outcome was known, and reporting bias must be considered, as there may have been outbreaks that were not reported to the PHU and therefore not included in this study. Potential bias due to variability in the degree of support provided to each facility by the PHU and RACF outreach teams is also a limitation.

Despite these limitations, and acknowledging that additional validation and replication is required, this study demonstrates a clear association between the IPC score and resident outcomes from COVID-19 outbreaks using real-world data. There is an opportunity for future research using patient level data to expand the sample size. This would allow exploration of associations between individual components of the IPC score and resident outcomes, and further adjustment for confounding factors in the analysis.

## Conclusion

While other rating systems such as the 5-star system have failed to consistently demonstrate an association with outcomes from COVID-19 outbreaks in RACFs [9], our unique IPC scoring system has demonstrated a promising association with attack rate and mortality rate during outbreaks of COVID-19 in RACFs. With further validation and replication of this scoring system, it could be used to assess facility preparedness for future outbreaks and allow health services to allocate resources accordingly to those facilities determined to be at an elevated risk of adverse outcomes.

### Supplementary Information

**Additional file 1:** **Appendix A.** IPC scoring matrix. **Appendix B.** Management scoring matrix.

## Data Availability

The datasets supporting the conclusions of this article are confidential and cannot be made publicly available for ethical reasons. For further information, please contact our corresponding author, Dr. George Johnson at George.johnson@health.nsw.gov.au.

## Abbreviations

COVID-19: Coronavirus Disease-19
GEE: Generalised Estimating Equations
IPC: Infection Prevention and Control
LHD: Local Health District
NSW: New South Wales
OMT: Outbreak Management Team
PCR: Polymerase Chain Reaction
PHU: Public Health Unit
PPE: Personal Protective Equipment
REDCap: Research Electronic Data Capture
RACFs: Residential Aged Care Facilities
RN: Registered Nurse
SARS-CoV-2: Severe Acute Respiratory Syndrome Coronavirus 2
SOP: Standard Operating Procedure
SLHD: Sydney Local Health District
WHO: World Health Organisation
