# Supplementary material for: Factors impacting resident outcomes from COVID-19 outbreaks in Residential Aged Care Facilities in Sydney Local Health District: testing an infection prevention and control scoring system
Source: BMC Public Health. 2023 Sep 11;23:1763. doi: 10.1186/s12889-023-16634-3 (PMC10494338; doi:10.1186/s12889-023-16634-3)
Supplement: Supplementary file 1 — Additional file 1: Appendix A. IPC scoring matrix. Appendix B. Management scoring matrix. [file 12889_2023_16634_MOESM1_ESM.docx]

**Appendix A: IPC scoring matrix**

| **RACF IPC and layout evaluation Tool** | | | | | | | | | | | | |
| --- | --- | --- | --- | --- | --- | --- | --- | --- | --- | --- | --- | --- |
| **Outbreak period:** | | **Number of zones:** | | **Date of IPC review:** | | | | | | | | |
| **RACF Layout evaluation** | **RACF layout evaluation tool** | | | | | | | | **Score** | | | **Tick** |
|  | **Care Type** | | | | | | | | | | | |
|  | High dependency (dementia) | | | | | | | | 3 | | |  |
|  | Moderate (aging in place) | | | | | | | | 2 | | |  |
|  | Low level care | | | | | | | | 1 | | |  |
|  | **Number of rooms** | | | | | | | | | | | |
|  | <100 | | | | | | | | 1 | | |  |
|  | >100 | | | | | | | | 2 | | |  |
|  | **Bathrooms to bed ratio** | | | | | | | | | | | |
|  | 1 | | | | | | | | 1 | | |  |
|  | 0.9-0.5 | | | | | | | | 2 | | |  |
|  | >0.5 | | | | | | | | 3 | | |  |
|  | **Ability to zone** | | | | | | | | | | | |
|  | 1-2 – low risk | | | | | | | | 1 | | |  |
|  | 3-4 – moderate risk | | | | | | | | 2 | | |  |
|  | 5-6 – high risk | | | | | | | | 3 | | |  |
|  | **Screening** | | | | | | | | | | | |
|  | Issues identified – *document issues* | | | | | | | | 1 | | |  |
|  | No issues identified | | | | | | | | 0 | | |  |
|  | **Mechanical ventilation** | | | | | | | | | | | |
|  | Issues identified – *document issues* | | | | | | | | 1 | | |  |
|  | No issues identified | | | | | | | | 0 | | |  |
|  | **Natural ventilation** | | | | | | | | | | | |
|  | Adequate air exchange | | | | | | | | 1 | | |  |
|  | Minimal air exchange | | | | | | | | 2 | | |  |
|  | **Meal distribution** | | | | | | | | | | | |
|  | High risk of contamination – refer to risks in … | | | | | | | | 2 | | |  |
|  | Low risk of contamination | | | | | | | | 1 | | |  |
|  | **Laundry** | | | | | | | | | | | |
|  | External | | | | | | | | 1 | | |  |
|  | Internal | | | | | | | | 2 | | |  |
|  | **Building structure** | | | | | | | | | | | |
|  | Built before | | | | | | | | 2 | | |  |
|  | Built after | | | | | | | | 1 | | |  |
|  | **Total** | | | | | | | | | | | /21 |
| **IPC Evaluation by ZONE** | **Zoning** | | **Point** | | **Zone 1** | **Zone 2** | **Zone 3** | **Zone 4** | | | **Zone 5** | |
|  | **Type of care** | | | |  |  |  |  | | |  | |
|  | High dependency and complex care (dementia, mental health) | | 3 | |  |  |  |  | | |  | |
|  | Moderate (aging in place) | | 2 | |  |  |  |  | | |  | |
|  | Low level care | | 1 | |  |  |  |  | | |  | |
|  | **Ability to isolate** | | | |  |  |  |  | | |  | |
|  | Positive residents cohorted | | 1 | |  |  |  |  | | |  | |
|  | No Cohorting of positive and negative residents | | 2 | |  |  |  |  | | |  | |
|  | **PPE** | | | |  |  |  |  | | |  | |
|  | TGA^1^ approved | | 1 | |  |  |  |  | | |  | |
|  | Not all items are TGA approved | | 2 | |  |  |  |  | | |  | |
|  | **Zone separation** | | | |  |  |  |  | | |  | |
|  | Ability to effectively separate zones | | 1 | |  |  |  |  | | |  | |
|  | Inability to effectively separate zones | | 2 | |  |  |  |  | | |  | |
|  | **Rooms** | | | |  |  |  |  | | |  | |
|  | Single rooms with ensuite | | 1 | |  |  |  |  | | |  | |
|  | Single rooms with shared bathrooms | | 2 | |  |  |  |  | | |  | |
|  | Double Rooms with Ensuite | | 3 | |  |  |  |  | | |  | |
|  | Quadruple rooms with ensuite | | 4 | |  |  |  |  | | |  | |
|  | **Nursing station** | | | |  |  |  |  | | |  | |
|  | Within zone | | 1 | |  |  |  |  | | |  | |
|  | Outside of zone | | 2 | |  |  |  |  | | |  | |
|  | **Treatment room** | | | |  |  |  |  | | |  | |
|  | Within zone | | 1 | |  |  |  |  | | |  | |
|  | Outside of zone | | 2 | |  |  |  |  | | |  | |
|  | **S8 medications** | | | |  |  |  |  | | |  | |
|  | Within zone | | 1 | |  |  |  |  | | |  | |
|  | Outside of zone | | 2 | |  |  |  |  | | |  | |
|  | **Entrance to facility** | | | |  |  |  |  | | |  | |
|  | Separate entrance | | 1 | |  |  |  |  | | |  | |
|  | Shared staff entrance | | 2 | |  |  |  |  | | |  | |
|  | Shared with staggered shift time | | 3 | |  |  |  |  | | |  | |
|  | **Exit of facility** | | | |  |  |  |  | | |  | |
|  | Separate entrance | | 1 | |  |  |  |  | | |  | |
|  | Shared staff entrance | | 2 | |  |  |  |  | | |  | |
|  | Shared with staggered shift time | | 3 | |  |  |  |  | | |  | |
|  | **Vaccination** | | | |  |  |  |  | | |  | |
|  | No unvaccinated residents | | 1 | |  |  |  |  | | |  | |
|  | ≥ 1 unvaccinated resident | | 2 | |  |  |  |  | | |  | |
|  | **Sub-total of zones:** | |  | | /27 | /27 | /27 | /27 | | | /27 | |
|  | **TOTAL:** | | | | | | | | | **/135** | | |
| **IPC Issues by ZONE** | **IPC issues identified throughout outbreak** | | **Point** | | **Zone 1** | **Zone 2** | **Zone 3** | **Zone 4** | | | **Zone 5** | |
|  | PPE | | 1 | |  |  |  |  | | |  | |
|  | PPE supply | | 1 | |  |  |  |  | | |  | |
|  | Donning/doffing stations | | 1 | |  |  |  |  | | |  | |
|  | Hand hygiene | | 1 | |  |  |  |  | | |  | |
|  | Waste management | | 1 | |  |  |  |  | | |  | |
|  | Cleaning | | 1 | |  |  |  |  | | |  | |
|  | Staff swabbing | | 1 | |  |  |  |  | | |  | |
|  | Resident swabbing | | 1 | |  |  |  |  | | |  | |
|  | Ventilation | | 1 | |  |  |  |  | | |  | |
|  | Signage | | 1 | |  |  |  |  | | |  | |
|  | Staffing | | 1 | |  |  |  |  | | |  | |
|  | Other | | 1 | |  |  |  |  | | |  | |
|  | Sub-total | | /12 | | /12 | /12 | /12 | /12 | | | /12 | |
|  | **TOTAL:** | | | | | | | | | **/60** | | |
|  | **Transmission rate (number of positive residents/total residents in facility)** | | | | | | | | | % | | |

^1^Therapeutic Goods Administration

**Appendix B: Management Scoring Matrix**

| **Concern Level** | **Concern Meaning** | **Primary Indication** | **Additional Indications** | **Escalation (if required)** |
| --- | --- | --- | --- | --- |
| 0 | No Concern | Clear delineation of competent management throughout the Facility and in attendance at OMTs | Clear involvement and engagement from Senior Management/Head office in Red Lockdown and OMT discussions. Clear delineated management structure in the facility. Clearly identified roles for each Red Lockdown task, including IPC Leads, Cohorted Registered Nurses (RNs), Line List Lead etc. | None required. |
| 1 | Some Concern | On site management but no involvement from Head Office and concerns regarding resourcing available to manage the Red Lockdown | Naivety from on site management regarding the resourcing and effort required to manage Red Lockdown. No indication of appropriate escalation to Head Office to assist in the management of the Red Lockdown. Concerns regarding a clear management structure and roles in place to manage aspects like IPC, RN support, Line listing etc. | Escalation to Head Office for involvement in OMTs and Red Lockdown. |
| 2 | Moderate Concern | Concerns regarding operational management of the Red Lockdown and support being provided by Head Office. | Clear incompetencies in operational management of Red Lockdown. Unwillingness, naivety, or incompetence from Head Office in ability to both support the management of the Red Lockdown and follow direction of the OMT. | SLHD Executive escalation to RACF CEO or Board regarding the management of the site. |
| 3 | Serious Concern | Significant concerns regarding management of Red Lockdown, clinical governance of residents and management of organisation. | Clear incompetencies with failure to improve management of Red Lockdown. Concerns regarding clinical governance and deterioration of residents. Clear indication that organisation is unwilling or incapable (operationally or financially) of supporting the Red Lockdown. | SLHD Executive escalation to Aged Care Quality Commission^a^ regarding management of the site, and consideration of requesting the facility to self-employ a Commonwealth Approved Nurse Advisor (only to occur following Step 2 escalation) |
| 4 | Urgent Concern Requiring Immediate Intervention | Clear indication of dangerous management practices and clinical incompetencies and deterioration of residents | Clear indication of systemic management incompetence. Clear clinical incompetencies introducing harm to residents’ care and wellbeing. Clear deterioration of residents. | CE written correspondence to Aged Care Quality Commission regarding urgent concerns regarding the management of the site requiring immediate intervention in the form of sanctioning and enforced employment of a Commonwealth Approved Nurse Advisor (only to occur following Step 3 escalation). |

^a^The Aged Care Quality Commission is the national regulator of aged care services in Australia.
